# Molecular Characterization of *Echinococcus granulosus* Sensu Lato from Livestock in North Khorasan Province, Iran

**Published:** 2018

**Authors:** Mitra SALEHI, Saeed YAGHFOORI, Pejman BAHARI, Mohsen SEYEDABADI, Sima PARANDE SHIRVAN

**Affiliations:** 1. Dept. of Parasitology, Faculty of Medicine, Gonabad University of Medical Sciences, Gonabad, Iran; 2. Student Research Committee, Gonabad University of Medical Sciences, Gonabad, Iran; 3. Vector-Borne Diseases Research Center, North Khorasan University of Medical & Laboratory of North Khorasan Veterinary Head office, Bojnurd, Iran; 4. Dept. of Pathobiology, Faculty of Veterinary Medicine, Ferdowsi University of Mashhad, Mashhad, Iran

**Keywords:** Cox1 gene, Genotypes, Hydatid cyst, Molecular epidemiology, Iran

## Abstract

**Background:**

*Echinococcus granulosus* is one the most important zoonotic disease which is endemic in worldwide. Molecular method has allowed discrimination of different genotypes (G1–G10), providing new approach in development of prevention and control program of hydatid cyst. This study was conducted to identify the genotypes of *E. granulosus* from domestic animals in nine districts of North Khorasan Province using the mitochondrial cox1 gene sequence.

**Methods:**

Overall, 122 hydatid cyst were collected during 2016–2017 from sheep (n=43) and cattle (n=79). DNA was extracted from protoscoleces and germinal layers and amplified by PCR. Phylogenetic analysis was also performed by analyzing the complete nucleotide sequences of mitochondrial cytochrome C oxidase subunit 1 (cox1) of *E. granulosus* genotypes from various locations.

**Results:**

Sequencing of the amplified products revealed the presence of G1 as dominant genotype, G3 and *Echinococcus canadenesis* in one isolate each. Altogether, 9 haplotypes were detected based on cox1 gene. Haplotype 3 was the common variant that found in 58 including 42 cattle and 16 sheep.

**Conclusion:**

This study provided knowledge on the identity of *E. granulosus* cysts collected from sheep and cattle in North Khorasan Province. Furthermore, these results showed the potentials of sheep as a main source of infection to humans, contributing the transmission and maintain of hydatid cyst in this region.

## Introduction

Cystic echinococcosis (CE), also known as cystic hydatid disease, is a severe zoonotic disease caused by the larval stages of Taeniid cestodes of *Echinococcus granulosus senso lato (s.l.). Echinococcus* spp. require two mammalian hosts to perpetuate their life cycle. Dog and other canids act as definitive hosts for adult worms, and ungulates serve as intermediate hosts for the cystic larva. Human is accidental intermediate host that become infected through ingestion of parasite eggs excreted by the feces of the infected dogs (1, 2). Despite the major control and prevention programs in reducing hydatid disease, this disease remains as a serious human and animal health concern (3).

CE is a cosmopolitan diseases that is endemic in many rural and pastoral areas of Asia (4–6). This disease is known to be endemic in many parts of Iran (7). Furthermore, CE has been reported with different prevalence (5% to 49%) in Iranian dogs (8–10). Human hydatidosis is responsible for about 1% of the surgical operation in Iranian hospitals (7, 11) and the incidence rate of this disease is reported to be 0.6–1.2 cases per 100000 inhabitants (7, 12), indicating high prevalence of CE in Iran.

Sheep-dog cycle is mainly present in Iran. Sheep and camel serve as the most important intermediate hosts (88% and 70%, respectively) (7, 13).

Recent molecular phylogenic analysis using mitochondrial genetic data have revealed 10 different genotypes for *E. granulosus* that differ in infectivity, host range and genetic characteristic (14). The following reconstruction based mainly on mitochondrial data of *E. granulosus s.l.* suggests four major species as follows: *E. granulosus sensu stricto* (*s.s*) (G1–G3), *E. equinus* (G4), *E. ortleppi* (G5) and *E. canadensis* (G6– G10) (15). “Camel and cattle strain cycles of *E. granulosus* require the shorter intervals for chemotherapy of dogs with respect to the shorter pre-patent period of these strains” (16). Therefore, knowledge of *Echinococcus* species involved in a region have benefits for the development of prevention and control programs and epidemiological studies (17).

An extensive body of evidence has indicated the high prevalence of CE in livestock and human in Iran (18). The annual economic loss incurred as a result of *hydatid cyst*-*related condemnation* of offal was estimated over U.S$219,349 in North Khorasan, where this study was conducted (19). Furthermore, surgical survey has been found evidence for the presence of human hydatidosis (20), considering the importance of molecular studying for elucidating the parasite epidemiology.

This study was conducted to extend the knowledge on molecular characterization of the larval stage of *E. granulosus* collected from sheep and cattle originating from North Khorasan Province, Iran.

## Materials and Methods

### Collection of hydatid cysts

Overall, 122 hydatid cysts were collected during 2016–2017 from slaughtered animals (sheep and cattle) during post-mortem inspection from various locations within North Khorasan Province, Iran. Collected cysts from lung and liver were placed in sterile saline solution and transported to the laboratory in ice box. To evaluate the cysts fertility, cyst contents were aseptically aspirated, centrifuged at 1500 gr for 10 min, and examined for the presence of protoscoleces. Protoscoleces were collected from fertile cysts, whereas germinal layers were collected from infertile cysts. Collected protoscoleces and germinal layers were washed several times in sterile saline and saved in −20 until DNA extraction.

### DNA extraction and Polymerase chain reaction (PCR)

Genomic DNA (gDNA) was extracted individually from the larval tissues of *E. granulosus* using a DNeasy blood and tissue kit (Qiagen, Germany) according to the manufacturer′s instructions and used as a template for polymerase chain reaction (PCR). Partial fragment of a mitochondrial gene for cytochrome c oxidase subunit 1 (cox1) was subjected to amplify by PCR using specific primers as described previously (21). PCR reaction was conducted in a 50 μl final volume containing 50–100 ng of gDNA, 200 μM of each dNTP, 3 mM of MgCl2, 10 pmol of each primer, and 1.5 U of Taq DNA polymerase. The DNA fragment of cox1 was amplified under following cycling condition, initial denaturation step of 94 °C for 5 min; 35 cycles of denaturation at 94 °C for 45 sec, annealing at 50 °C for 45 sec and extension at 72 °C for 45 sec; followed by a final extension at 72 °C for 10 min. The resulting amplicons from each PCR were analyzed through *1.5*% *agarose gel* electrophoresis and were visualized by ethidium bromide staining under UV.

### DNA sequence analysis

Amplified products were commercially purified and sequenced using the forward primer employed for PCR (Bioneer, South Korea). The quality of the sequences was evaluated and edited by BioEdite software 7.0.5 (22) and then compared to those available in the Gen-Bank database using BLAST sequence algorithms to determine the genotype of *Echinococcus* isolates (https://blast.ncbi.nlm.nih.gov/Blast.cgi).

All 122 nucleotide sequences obtained in the present study were deposited in GenBank under accession numbers KR733081-KR733083-88, KR920697, KR920700-701, KT200218-20, KT200222-23, KT254111-19, KT254121-25, KT320877-88, KU360296-325, KU603673-79, KU603681-707, KU603709-726, and KU603728-729 for cox1 sequences.

### Phylogenetic analysis

A phylogenetic analysis based on the haplotype approach was conducted to estimate the similarity/distance of parameters. In brief, previously published sequences of different *E. granulosus* isolates were used as reference sequences (Table 1). Nucleotide data including reference sequences and haplotypes sequences from this study were aligned with the Clustal W (23) algorithm using BioEdit version 7.0.5 (22). The HKY + gamma + T model was selected as the best fit model using j model test 0.1.1 software (24). The selected model based on the Akaike Information Criterion was applied to construct phylogenetic relationships between the haplotypes using the Maximum likelihood tree as implemented in PAUP 4.0b10 (25). Reliability of internal branches was evaluated using non-parametric bootstrapping with 1000 replicates. *Taenia saginata* was included as outgroup.

**Table 1:** Accession number and geographical locations of *Echinococcus* cox1 sequences used in the present phylogenetic analysis

***Genotype***	***Host***	***Accession number***	***Country***
G1	Cattle	HM636639	Lybia
G1	Sheep	HQ717149	Turkey
G1	Sheep	DQ856467	Greece
G1	Human	JX854034	India
G1	Sheep	HM563001	Iran (Kerman)
G1	Goat	HM563010	Iran (Kerman)
G1	Dog	JN604097	Iran (Lorestan)
G1	Sheep	JF775380	Turkey
G2	Sheep	M84662	Tasmania
G2	Dog	JN604103	Iran (Lorestan)
G2	Goat	KJ162562	Iran (Kashan)
G3	Sheep	DQ856466	Greece
G3	India	JX854028	India
G3	Sheep	HM563016	Iran (Kerman)
G3	Buffalo	M84663	India
G3	Dog	JN604104	Iran (Lorestan)
G4	Horse	M84664	Spain
G5	Camel	AB921055	Egypt
G5	Human	JX854035	India
G5	Cattle	AB235846	Argentina
G5	Cattle	M84665	Holland
G6	Camel	NC011121	Kazakhstan
G6	Camel	AB921058	Egypt
G6	Camel	AB921084	Egypt
G6	Camel	HM563018	Iran (Kerman)
G6	Camel	M84666	kenya
G6	Human	KC415063	India
G6	Camel	HM856354	Iran
G7	Human	KJ556997	China
G7	Pig	M84667	Poland
G8	Moose	AB235848	USA
G10	Reindeer	AF525457	Filand
G10	Human	KJ663947	China
G10	Moose	AB777911	Russia
Taenia saginata	Human	AB465246	South Korea

### Sequence homology

Haplotype segregation in obtained sequences in the present study was performed by DnaSP software version 5 (26). Multiple alignments of sequence information using Clustal W estimated the extent of variation in detected genotype by pairwise comparison of haplotype sequences with each other and reference sequences. To determine the synonymous and non-synonymous substitution, the nucleotide sequences translated into the corresponding amino acids using CLC genomics software version 9 (CLC bio, Aarhus, Denmark).

## Ethical aspects

All samples were collected post-mortem in the slaughterhouse and caused no suffering to the animals.

## Results

The examination of organ distribution of CE indicated pulmonary and hepatic cysts in both animals. In cattle, lung was more likely to be infected than liver, 64.55%, and 35.44%, respectively. While liver and lung cysts were equal in sheep (22 vs 21). The fertility rates of hydatid cysts were 78% and 12.22% in sheep and cattle, respectively.

Pulmonary cysts had higher fertility than liver cysts in both sheep and cattle. The highest rate of fertility was determined in pulmonary cysts of sheep (80.95%), and the lowest in cattle’s liver (10.3%).

### Molecular analysis

All genomic DNA samples derived from individual *E. granulosus* cysts were subjected to PCR of cox1 gene. Successful PCR amplification of cox1 gene yielded amplification product of 446 base pair. Single bands on agarose gel indicated the specificity of the PCR protocol employed. The obtained consensus haplotype sequences of cox1 were 304 bp. Alignments of the obtained sequences derived from sheep isolates indicated the existence of G1 genotype (sheep strain) in 42 of 43 isolates and *E. canadensis* in one isolate. Totally, 78 of 79 cattle were infected with G1 (*E. granulosus* sensu stricto), and the remaining one with G3 (buffalo strain).

### Phylogenetic analysis

**Fig. 1:**
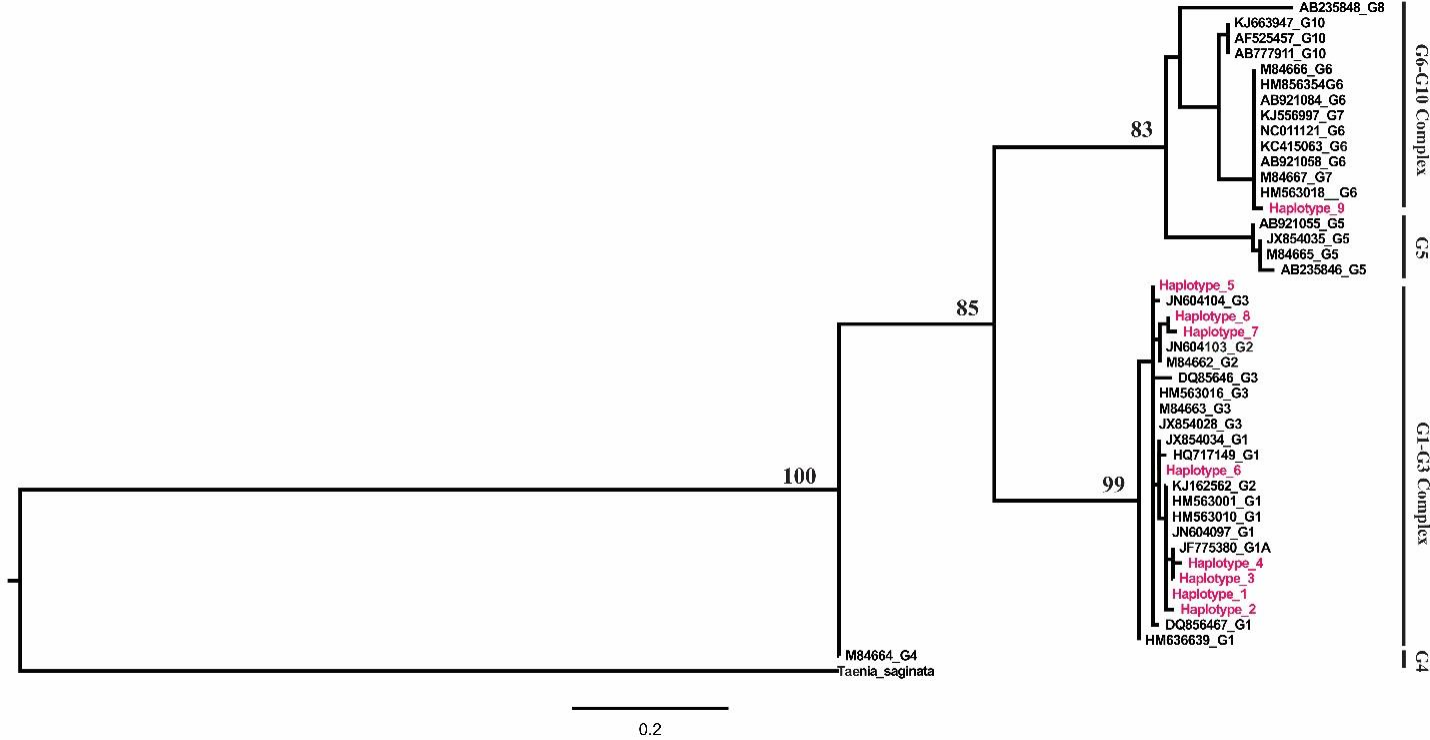
Phylogenetic relationships among obtained haplotypes in this study and reference sequences retracted from NCBI. The phylogenetic tree was constructed on COX1 sequences using the Maximum likelihood algorithm as implemented in PAUP 4.0b10. *Taenia saginata* served as outgroup. The scale bar represents distance

**Fig. 2:**
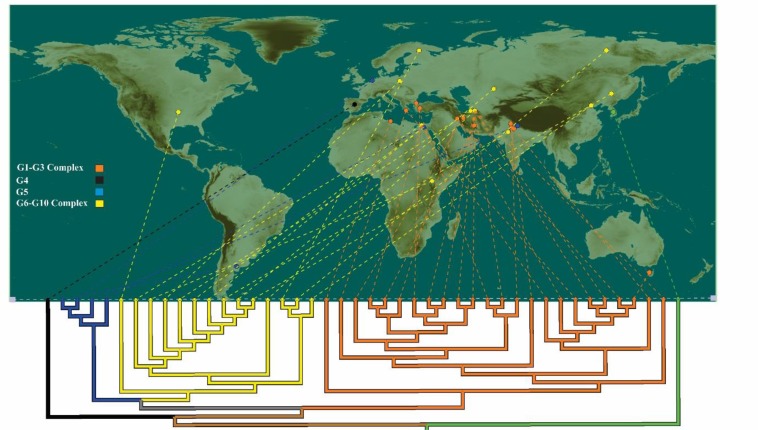
Phylogeography of *E. granulosus* species. GenGIS software was used to represent a clear view of the relationship between geography and genomic diversity. Each of the four genotypes within *E. granulosus* is assigned a unique color (G1–G3: orange, G4: black, G5: blue and G6–G10: yellow). A sequence of *Taenia saginata* as the correspondent outgroup sequence is identical by green color

Phylogenetic analysis of cox1 sequences revealed four main clades including the previously well-known G1–G3 complex, G4, G5 and G6–G10 complex. G5 (*E. ortleppi*) formed a sister phylogenetic group with G6–G10 complex. G4 was distinct from other *E. granolossus* genotypes (G1–G3, G5, and G6–G10). Totally from 9 haplotypes detected in cox1 sequences, 8 haplotypes grouped with reference sequences from G1–G3 complex, particularly G1. Haplotype 9 clustered with G6 and G7 genotypes, separating from G10 genotypes. Intra-group genetic variation observed in all main groups. Maximum likelihood analysis of the 9 haplotypes along with reference sequences was shown in Fig. 1. Furthermore, the integration of the phylogenetic tree with geographical information from reference sequences used in this study was represented in Fig. 2.

### Sequence polymorphism in COX1 gene

The alignment of the cox1 sequences indicated 9 different haplotypes (including 7 G1s, one G1–3, and one *E. canadensis*). Among all 9 haplotypes, haplotype 3 was the common variant, found in 58 isolates including 42 cattle and 16 sheep. Haplotype 1 was the second current variant with 42 isolates including 17 sheep and 25 cattle. The other haplotypes (7 haplotypes) observed in 22 isolates (Table 2).

**Table 2:** Accession number for the partial cox 1 sequences derived from this study

***Haplotype (Genotype)***	***Host origin (number)***	***Accession numbers***					
Haplotype 1 (G1)	Sheep (17), Cattle (25)	KU603689	KU603713	KU360312	KU603718	KU603717	KU603716
		KU603703	KU603711	KU603678	KU603712	KU360301	KU603723
		KT320879	KT254121	KT254117	KT320881	KT320882	KT254111
		KU360298	KU360302	KU360296	KT254114	KT254125	KU603679
		KU603673	KT200220	KT254116	KU603675	KU360308	KU603685
		KU603719	KU603707	KU603726	KT320888	KU603725	KU603722
		KR920697 KU603715 KU603721 KT320883 KT320884 KT320887					
Haplotype 2 (G1)	Sheep (1), cattle (4)	KU360316 KU603709 KU603693 KU603728 KT320877					
Haplotype 3 (G1)	Sheep (16), Cattle (42)	KU360297	KU360304	KU603681	KR733081	KU360323	KU603700
		KU360306	KU360300	KU360319	KU603683	KU360324	KU603677
		KU360303	KU603701	KU360322	KR733088	KT254123	KU603692
		KU603704	KU603699	KU360307	KU360314	KU360310	KU603684
		KU603691	KU603688	KU360311	KU603682	KU603714	KU603729
		KT320880	KT320878	KU603710	KU603686	KU603690	KU603698
		KT254122	KR920700	KU360320	KT254119	KU603697	KU360321
		KT254124	KU603706	KT254113	KU603705	KU360305	KU360313
		KU603674	KU360325	KU603702	KT200219	KU603687	KT254118
		KU360309 KU360315 KT200218 KT200223					
Haplotype 4 (G1)	Sheep (1), cattle (2)	KT200222 KT254112 KT254115					
Haplotype 5 (G3)	Cattle (1)	KR733086					
Haplotype 6 (G1)	Sheep (4), cattle (3)	KU360299	KU603676	KU603695	KU603696	KU360318	KR920701
		KT320885					
Haplotype 7 (G1)	Sheep (1), cattle (2)	KT320886 KU603720 KU603724					
Haplotype 8 (G1)	Sheep (2)	KU603694 KU360317					
Haplotype 9 (G6)	Sheep (1)	KR733084					

**Fig. 3:**
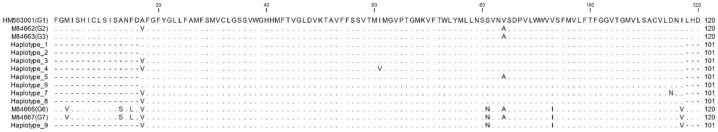
ClustalW alignments of partial cox1 amino acid sequences. Accession numbers HM563001, M84662, M84663, M84666, and M84667 represent G1, G2, G3, G6 and G7 reference sequences, respectively

The alignment of the cox1 sequence indicated intra-genotype sequence variation within G1 and G6 genotypes (Fig. 3). Haplotype 1 showed complete identify (100%) to G1 reference sequence (HM563001). Haplotype 2 had a single nucleotide substitution of C to T at position 105 as compared to reference sequence HM563001, but this substitution was synonymous. Synonymous substitution was also observed in haplotype 6 with a transition of C to T at position 13. Haplotype 3 showed a nucleotide change of C to T at position 3, leading to non-synonymous substation of Alanine to Valine. Two variable non-synonymous substitutions were observed in haplotype 4, one substitution (C to T) at position 3 led to substitution of Alanine to Valine and the other (A to G) at position 134, causing transition of Isoleucine to Valine. Comparison of G1 reference sequence (HM563001) and haplotype 5 showed two differences.

One substitution at the 204 positions (T to C), leading to substitution of Valine to Alanine and the other at the 13 positions, causing non-synonymous substitution. On the other hand, this haplotype generated a sequence with 100% identify to G3 reference sequence (M84663). Haplotype 7 had the most sequence variation with one synonymous (C to T at position 13) and two non-synonymous substitutions. Nucleotide substitution (G to A) at position 295 changed Aspartate to Asparagine. Substitution of Alanine to Valine was generated by transition of C to T at position 3. Haplotype 8 had also both non-synonymous and synonymous substation at position 3 and 13, respectively. Haplotype 9 showed 99% identify to G6 and G7 reference sequences (M84666) with a single transition (C to T) at position 204, leading non-synonymous substitution (Alanine to Valine).

## Discussion

In the present study, three genotypes were identified to infect cattle and sheep: G1, G3 and *E. canadensis.* G1 was found as the most common genotype in North Khorasan Province, consistent with the earlier study from Iran (27, 28). The presence of G1 was found in all thirty liver and lung samples from cattle, sheep, and goats of abattoirs in northern and western Iran using DNA sequences of the mitochondrial 12S rRNA gene (29). A predominance of G1 with a small number of G3 using cox1 gene was showed in five different provinces of Iran (30). In contrast, a study on 19 camel hydatid cysts collected from central Iran revealed the majority of G3 genotype in isolates (31). The dominant of G1 over the other genotypes was also reported from other countries: such as China (32), Turkey (33) and Southern Brazil (34). The occurrence of both sheep strain (G1) and buffalo strain (G3) have been demonstrated in different intermediated host in Iran (35, 36) and other countries (34, 37). For example, the presence of G3 genotype was found in 3 cattle and 2 sheep along with the majority of G1 in both animals (107 isolates) (38). A similar finding was reported in Italy on 80 cattle and water buffalos (78.75% G1 vs. 12.5% G3) (39). In contrast, G3 and G6 were the dominant genotypes in India (40, 41) and Egypt (42, 43), respectively. Considering that sheep strain (G1) was the most frequent genotype, it seems to sheep-dog cycle was responsible for establishment and maintenance of *Echinococcus* life cycle in North Khorasan Province where this study was conducted. However, G3 and G6 genotypes are known human pathogens and should be considered as a significant public health concern.

In the present study 9 haplotypes were identified based on the alignment of Cox1 sequences. In comparison with our result, haplotype segregation of previous studies from other provinces in Iran showed a higher diversity of *E. granulosus* sensu stricto (G1–G3). For example studies in Ardabil (44), Lorestan (8) and Zanjan province described 13 haplotypes (35). This difference may be related to the length of the gene analyzed, province of study and sample size. The outcomes of haplotype segregation could be affected by the length of the gene analyzed (45).

In this study, the topology of *Echinococcus* clade from this tree was consistent with previous studies (46–48). The present phylogeny based on maximum likelihood supported the validity of the G1–G3 complex to distinct from other genotypes and withhold that G2 was a distinct genotype. Moreover, the tree showed a monophyly of *E. ortleppi* and *E. canadensis* and supported the nation that *E. canadensis* are closely related to each other. The tree topology suggested that G10 and G8 were paraphyletic and G10 was sister to G6 and G7. Our results provided supportive evidence for the revision of genotype G4 into *E. equinus* (17). In our phylogenetic tree haplotypes, 1–8 grouped with published sequences representing genotypes G1–G3. Haplotype 9 placed in a close genetic relatedness of G6 and G7. Considering that G6 and G7 are descendants of a common ancestor (47) and based on our phylogenetic analysis, the name of *E. canadensis* seems to be the most suitable for haplotype 9. The alignment of haplotype 9 along with G6 (M84666) and G7 reference sequences (M84667) was also showed 99% identify to both sequences, supporting phylogenetic results. Haplotype 5 grouped into G1–G3 complex with the most close related to G3 genotype.

## Conclusion

The *Echinococcus* genotypes identified in this study, G1, G3, and G6, are known human pathogen, exerting significant public health concern. Molecular analysis showed the presence of G1 (sheep strain) as the prominent genotype of *Echinococcus* in sheep and cattle in North Khorasan Province of Iran. Considering the presence of poor rural communities where people and livestock are in close contact to dog, prevention and control program should be imposed on sheep – dog cycle. Although cattle were found to be infected with G1, they did not contribute to the transmission of the disease because most of the cysts were sterile.

Although this study has provided a glimpse of the genotypes of *E. granulosus* in North Khorasan Province, a large study is needed to investigate the isolate from different hosts and from multiple geographic areas to better understand the transmission and epidemiology of different genotypes in Iran.
